# No Evidence of Persisting Unrepaired Nuclear DNA Single Strand Breaks in Distinct Types of Cells in the Brain, Kidney, and Liver of Adult Mice after Continuous Eight-Week 50 Hz Magnetic Field Exposure with Flux Density of 0.1 mT or 1.0 mT

**DOI:** 10.1371/journal.pone.0109774

**Published:** 2014-10-10

**Authors:** Hubert Korr, Nicholas B. Angstman, Tatjana B. Born, Kerstin Bosse, Birka Brauns, Martin Demmler, Katja Fueller, Orsolya Kántor, Barbara M. Kever, Navida Rahimyar, Sepideh Salimi, Jiri Silny, Christoph Schmitz

**Affiliations:** 1 Department of Anatomy and Cell Biology, RWTH Aachen University, Aachen, Germany; 2 Department of Neuroanatomy, Ludwig-Maximilians-University of Munich, Munich, Germany; 3 Department of Anatomy, Histology and Embryology, Semmelweis University, Budapest, Hungary; 4 Femu, Institute of Occupational and Social Medicine, RWTH Aachen University, Aachen, Germany; Montana State University, United States of America

## Abstract

**Background:**

It has been hypothesized in the literature that exposure to extremely low frequency electromagnetic fields (50 or 60 Hz) may lead to human health effects such as childhood leukemia or brain tumors. In a previous study investigating multiple types of cells from brain and kidney of the mouse (Acta Neuropathologica 2004; 107: 257–264), we found increased unrepaired nuclear DNA single strand breaks (nDNA SSB) only in epithelial cells of the choroid plexus in the brain using autoradiographic methods after a continuous eight-week 50 Hz magnetic field (MF) exposure of adult mice with flux density of 1.5 mT.

**Methods:**

In the present study we tested the hypothesis that MF exposure with lower flux densities (0.1 mT, i.e., the actual exposure limit for the population in most European countries, and 1.0 mT) shows similar results to those in the previous study. Experiments and data analysis were carried out in a similar way as in our previous study.

**Results:**

Continuous eight-week 50 Hz MF exposure with 0.1 mT or 1.0 mT did not result in increased persisting unrepaired nDNA SSB in distinct types of cells in the brain, kidney, and liver of adult mice. MF exposure with 1.0 mT led to reduced unscheduled DNA synthesis (UDS) in epithelial cells in the choroid plexus of the fourth ventricle in the brain (EC-CP) and epithelial cells of the cortical collecting duct in the kidney, as well as to reduced mtDNA synthesis in neurons of the caudate nucleus in the brain and in EC-CP.

**Conclusion:**

No evidence was found for increased persisting unrepaired nDNA SSB in distinct types of cells in the brain, kidney, and liver of adult mice after continuous eight-week 50 Hz magnetic field exposure with flux density of 0.1 mT or 1.0 mT.

## Introduction

It has been hypothesized in the literature that exposure to extremely low frequency electromagnetic fields (50 or 60 Hz) may lead to human health effects such as childhood leukemia or brain tumors [1]. However, this hypothesis was derived from epidemiological studies which *per se* do not implicate causal relationships. The latter can only be addressed with experiments carried out under carefully controlled conditions. Among the experiments on rats and mice listed in the ‘BioInitiative Report’ [1], the following results related to brain cells seem to be of particular importance: (i) Lai and Singh [2]–[4]; (see also [5]) found nuclear DNA single-strand breaks (nDNA SSB) and double-strand breaks (DSB) from 0.01 mT magnetic field (MF) exposure onwards in a dose-dependent manner in rats. It is of note that these effects could be blocked by pretreating rats with a vitamin E analog, a nitric oxide synthase inhibitor, or an iron chelator. From these data, the authors concluded that MF exposure might lead to increased generation of free radicals via the so-called Fenton reaction within mitochondria which, thereafter, cause nuclear DNA damage. (ii) Schmitz et al. [6] showed that continuous 50 Hz MF exposure with flux density of 1.5 mT over 8 weeks led to increased nDNA damage (probably unrepaired nDNA SSB) exclusively in epithelial cells of the choroid plexus of the fourth ventricle in the mouse brain, i.e., a small group of cells involved in the production of cerebrospinal fluid (CFS) and, notably, in iron transport from blood into the brain interstitium [7]. This iron transport is connected to the production of free radicals via the Fenton reaction [8]. Schmitz et al. [6] therefore hypothesized that MF exposure mainly affects iron transport, potentially causing increased nDNA damage in the affected cells.

Accordingly, one could conclude that MF exposure may lead to nDNA damage via the generation of free radicals. However, the question remains open as to whether this effect is present in all brain cells (due to an increased production of free radicals in their mitochondria) or preferentially in a relatively small group of cells which are involved in iron transport. In the brain, plexus epithelial cells and endothelial cells (bordering the blood vessels and being part of the so-called blood-brain barrier) transport iron into the liquor or the brain [9]. With respect to potential consequences, however, damage related to all cells (due to the presence of mitochondria) may be less dangerous in the long run (probably because of rapid damage repair) than damage only to a distinct group of cells. In this regard, the epithelial cells of the choroid plexus seem to be of particular importance. These cells are defined as a subtype of macroglia (for comprehensive reviews see [10], [11]). In addition to CSF production, the choroid plexus acts as a filtration system, removing metabolic waste and excess neurotransmitters from the CSF [11]. Hence, the epithelial cells of the choroid plexus have an important role in helping to maintain the extracellular environment required by the brain to function optimally. The choroid plexus is involved in a variety of neurological disorders, including inflammatory, infectious, neurodegenerative, and neoplastic diseases [10], [11]. For example, amyloid beta accumulates in the choroid plexus in Alzheimer's disease [10]. Furthermore, choroid plexus papilloma and carcinoma represent the most common brain tumors in the first year of life [10].

A conclusive answer to the question raised above can only be given when methods are used that allow cell type specific analyses *in situ*. This is not the case when demonstrating DNA strand breaks with the so-called comet-assay (single-cell gel electrophoresis; see, e.g., [12]), even though this method is very sensitive when used properly [5]. Rather, cell type specific effects can be measured with the following specific autoradiographic methods (c.f. [6]): (i) *in situ* nick translation (ISNT), representing the relative amount of unrepaired nDNA SSB at the time of an animal's death [6], [13], [14]; and (ii) unscheduled DNA synthesis (UDS), demonstrating a preceding nDNA repair [14]. Concerning the sensitivity of these methods, one has to take into account that autoradiographic silver grains seen over a histologic structure are the product of ^3^H-radioactivity present in the structure (e.g., the nucleus), as well as the exposure time of the autoradiograph. The latter can be increased up to 12 months, provided that very specific technical prerequisites are given [15]. This is due to the fact that the photographic emulsion used in this procedure (Ilford K2; Ilford, Mobberley, UK) works linearly over a time interval of 12 months [16]. With an exposure time of 250 days, silver grains produced by less than one beta decay per cell nuclear profile area per day could be reproducibly obtained in previous UDS studies [6], [13], [17], [18]. In the case of ISNT studies, only indirect information regarding sensitivity has been reported: Wang et al. [19] showed a linear relationship between the number of cell nuclear silver grains and dose for some cell lines *in vitro* after gamma irradiation in the range of 0–1 Gy. It appears feasible that enhanced sensitivity could have been obtained when increasing the exposure time of one day used by the authors of this study.

Autoradiographs of UDS studies can also be evaluated with respect to cytoplasmic grain densities, representing mitochondrial (mt) DNA synthesis rates at the time of ^3^H-TdR application (for details see [6], [13], [20], [21]).

In order to obtain more information about the effects of MF exposure on DNA *in situ* in a cell type-specific manner, we carried out further experiments using a very similar experimental setup and mode of evaluation as described previously [6]. The following questions were addressed: (i) Does MF exposure with much lower flux density than 1.5 mT (applied in [6]) also result in significant effects on DNA? (ii) If this is the case, are all cells or only specific cell types affected (such as the epithelial cells of the choroid plexus engaged in iron transport)? (iii) How do cells that are highly involved in iron storage react to MF exposure?

Experiments were carried out on mice with MF flux density of 0.1 mT at 50 Hz as this value represents the current exposure limit for the general population in most European countries [22]. In order to study dose-dependent effects, we also exposed mice to 1.0 mT at 50 Hz. Alongside the brain and kidney (where the epithelial cells of all tubular and duct segments are involved in reabsorption of iron [23]), we also analyzed liver cells strongly involved in iron storage.

## Methods

### Animals

A total of n = 86 male outbred mice (strain Hannover (Han): Naval Medical Research Institute (NMRI); 6 weeks old) were obtained from Charles River Lab (Sulzfeld, Germany). These mice (up to five brothers per cage) were kept under constant, specific pathogen free (SPF) conditions (20°C, 60% humidity, artificial light from 6.00 a.m. to 6.00 p.m., fed with Altromin standard diet and water *ad libitum*). At the animals' age of seven months, MF exposure (n = 60) was started in a separate room under the same settings except for SPF conditions. During MF exposure, animals were kept in metal-free plastic cages. Every second day the cages were exchanged for cleaning without interruption of the MF exposure. Unexposed control mice (n = 26) were kept under identical conditions in the same room as the MF exposed animals, but outside of Helmholtz coils (as described below). The distance between the cages of the MF exposed and unexposed mice was greater than 3 m.

### Ethics statement

All experiments were approved by the Animal Ethics Committee of the District Government in Cologne, Germany.

### 50 Hz Magnetic Field Exposure

**Figure 1:**
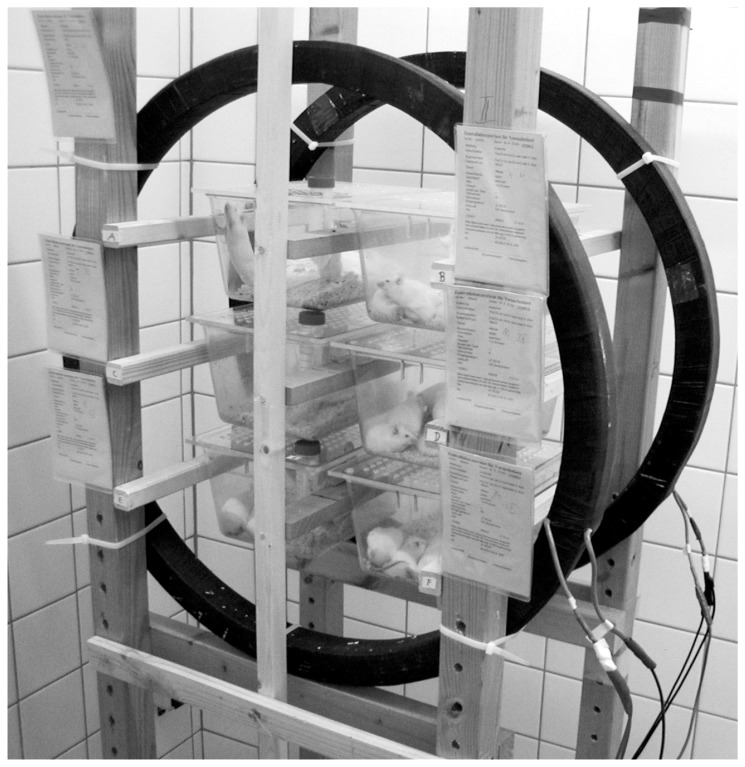
Experimental set-up of the 50 Hz MF exposure of the present study. Details are provided in the main text.

Two groups of n = 30 mice, each located in six cages of 14 cm height, 16 cm width, and 22 cm depth, were simultaneously exposed to a fairly uniform horizontally oriented sinusoidal magnetic 50 Hz field (MF) with a magnetic flux density of 0.1 mT (group A) or 1.0 mT (group B) (Fig. 1; note that the experimental set-up used in the present study differed from the experimental set-up used in our previous study [6], in which we exposed a smaller number of mice to a magnetic flux density of 1.5 mT). Due to the movement of the animals in the cages, the orientation of the 50 Hz MF had no influence on the outcome of the present study (which is in line with the literature). For the generation of magnetic fields, a pair of identical circular Helmholtz coils with a diameter of 69 cm and separated by a distance of 34.5 cm were used (Fig. 1). The coils were fed from a main supply (220 V, 50 Hz) by an adjustable transformer (Philips type 2422, Eindhoven, Netherlands). The electric current, flowing in the same direction in each coil, was adjusted by an RMS Clamp Meter 33 (Fluke Corporation, Everett, WA, USA) to produce a magnetic flux density of 0.1 mT or 1.0 mT at the center of the setup. Measurements of the alternating magnetic fields both in the area between the Helmholtz coils and in the laboratory, were carried out with an electromagnetic meter (EM 400; Symann & Trebbau, Lippstadt, Germany). Within the Helmholtz coils, the flux density between the center and the periphery varied no more than 7%. The Helmholtz coils were electrically shielded, and the electric field strength of the 50 Hz field in the exposed cages was below 10 V/m. The distortion factor of the current in the coils, measured with a Distortion Measurement Set 339 A (Hewlett Packard, Loveland, CO, USA), remained below 2%. The magnetic fields generated in the laboratory by the 50 Hz power supply were lower than 0.2 µT. In the control cages, the maximal flux density of 50 Hz MF was 0.15 µT. The earth's DC magnetic field, measured by a RFL Gauss meter 912 (RFL Electronics, Boonton Township, NJ, USA), was below 50 µT in all areas of the investigations.

### Injection of ^3^H-TdR and Perfusion Fixation

Mice were divided into three groups. Groups A and B were MF exposed (A: 0.1 mT, n = 30; B: 1.0 mT, n = 30); mice in group C were unexposed (n = 26). After an eight week interval with or without 50 Hz MF exposure, 10 mice each group—stemming from different cages—were randomly selected and brought to another room where they received an intraperitoneal injection of 555 kBq [methyl-^3^H] thymidine (^3^H-TdR; 2,664 GBq/mmol; American Radiolabeled Chemicals, St. Louis, MO, USA) per g body mass (b.m.) within 5 min of removing the animals from the MF exposure. Each mouse was anaesthetized with chloral hydrate (10% aqueous solution, 0.005 ml/g b.m., i.p.) 115 min after ^3^H-TdR injection and killed 5 min later (i.e., two hours after ^3^H-TdR injection) by intracardial perfusion with the following solutions in a consecutive manner (total perfusion time 3–4 min per animal; perfusion via the left ventricle with the inferior vena cava opened): (i) 20 ml Thomaedex (Thomae, Biberach, Germany); (ii) 20 ml formalin: 0.9% NaCl (1∶9; pH 7.2); (iii) 30 ml formalin: 0.9% NaCl (1∶4) to which acetic acid was added (1%; [24]); and (iv) 20 ml formalin: 0.9% NaCl (1∶4; pH 7.2). To determine autoradiographic background and cell-type-specific chemography (see below), two of the unexposed mice were sacrificed in the described manner but without injection of ^3^H-TdR. The remaining mice (Groups A and B: n = 20 each; Group C: n = 16) were not investigated in the present study.

From each perfused animal, the head, part of the liver (left lobe), and one kidney were dissected. After opening the skulls without removing the brain, the heads were fixed in formalin solution no. iv for 2–6 h at 4°C. Afterwards, the brains were removed, halved at the mediosagittal line, and further fixed in formalin solution no. iv for two weeks. The other organs were also post-fixed in formalin solution no. iv for two weeks. All organs and the right brain halves were embedded in paraffin.

### Preparation of Autoradiographs

3 µm-thick sections (paramedian, sagittal sections in case of the brains) were cut with a motor-driven rotation microtome (Leica RM 2065, Leica Instruments, Nußloch, Germany) using 25-mm Ralph type glass knives prepared by an LKB 2078 HistoKnife Maker (LKB Produkter, Bromma, Sweden).

For studying UDS and mtDNA synthesis, sections were mounted on low potassium slides (Kindler, Freiburg, Germany), deparaffinized with xylol, and Feulgen stained (1N HCl at 60°C for 6 min). The slides were dipped into diluted Ilford K2 emulsion (1∶1 with distilled water; Ilford, Mobberley, UK) at 42°C, exposed 250 days (with exception of liver sections, which were only exposed for 125 days) at 4°C, and developed in Amidol (4 min, 18°C). After post-staining with 0.5% Light Green SF Yellowish (Merck, Darmstadt, Germany), the slides were covered with coverslips using Entellan (Merck).

For studying unrepaired nDNA SSB by ISNT (carried out on the same mice used for the analysis of UDS and mtDNA synthesis), 3 µm-thick paraffin sections were mounted on slides coated with 3-aminopropyltriethoxysilane (APES, Sigma-Aldrich, St. Louis, MO, USA) for improved adhesion [25]. After removing paraffin with xylol, the sections were treated with a solution (Tris-HCl-buffer, pH 8, and 5 mM ethylenediaminetetraacetic acid (EDTA)) containing 25 µg proteinase K (Roche Diagnostics, Mannheim, Germany) per ml. Subsequently, ISNT was carried out as follows [6], [13]: sections were incubated for 60 min at 37°C with a reaction solution containing 50 mM Tris-HCl-buffer (pH 7.5), 5 mM MgCl_2_, 10 mM 2-mercaptoethanol, 200 U/ml of *Escherichia coli* DNA polymerase-I (endonuclease-free; Roche), 10 µmol/ml each of dATP, dGTP, dCTP, and dTTP (Sigma-Aldrich), and 15 µl ^3^H-dTTP/ml (1.03 MBq/ml; American Radiolabeled Chemicals). Incubation was carried out using special *in situ* chambers consisting of small, custom-made frames of silicon (height  = 1 mm; comparable to customary adapters for *in situ* polymerase chain reaction) and coverslips placed onto the slides. The reaction was terminated by washing the slides with 50 mM Tris-HCl buffer (pH 7.5). Afterwards, sections were Feulgen stained for cell identification according to their nuclear morphology, as well as for removing non-incorporated ^3^H-dTTP. Then, the slides were dipped into diluted Ilford K2 emulsion (1∶1 with distilled water) at 42°C, exposed for 7–12 days at 4°C, and developed in Amidol (4 min, 18°C). After post-staining with 0.5% Light Green SF Yellowish (Merck), the slides were covered with cover slips using Entellan (Merck).

### Evaluation of Autoradiographs

The following types of cells were analyzed: (i) neurons in the caudate nucleus of the brain; (ii) epithelial cells in the choroid plexus of the fourth ventricle in the brain; (iii) epithelial cells of the cortical collecting duct in the kidney, and (iv) pericentral hepatocytes in the liver (i.e. cells lying around a central vein with the exception of hepatocytes directly adjacent to a vein). When studying nDNA damage and its repair in non-proliferating hepatocytes, two criteria must be taken into consideration: (i) their grade of polyploidization, representing different levels of metabolic activity; and (ii) their position within the liver lobule, determining their role in the process of detoxification of the venous blood [26]. In the present study, only 2n hepatocytes were evaluated—identified on the basis of their nuclear profile areas (according to [27]). Based on data from a previous study of our group, hepatocytes with a profile area between 18.6 µm^2^ and 48 µm^2^ were considered diploid [28].

**Figure 2:**
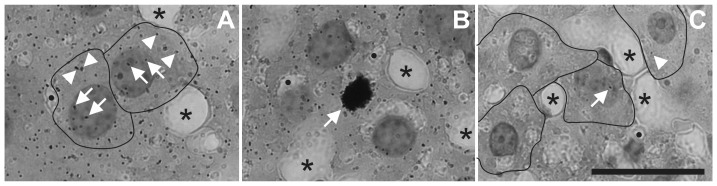
Representative autoradiographs of Feulgen-prestained and Light Green SF Yellowish post stained paraffin sections analyzed in the present study. The photomicrographs show details of the mouse liver after an 8-week 50 Hz MF exposure with 1.0 mT with (A, B) or without (C) injection of ^3^H-TdR 5 min after the end of the MF exposure. Both autoradiographs were exposed in the same box for 125 days, i.e. under completely identical conditions. In A, a few (out of many) individual silver grains found over the nucleus (arrows) and cytoplasm (arrowheads) of two hepatocytes are marked; the cell bounds are indicated. In B, the arrow points to a cell that was in S phase after injection of ^3^H-TdR (i.e., during the last two hours of life). In C, single silver grains found over the nucleus (arrow) or cytoplasm (arrowhead) of hepatocytes are marked, representing autoradiographic background. In all panels A to C, black dots and asterisks indicate small and larger sections of liver sinusoids, respectively. The scale bar represents 25 µm.

The analyses were independently performed by eight researchers on coded autoradiographs; i.e., the researchers did not know whether the coded autoradiographs belonged to an MF exposed or an unexposed control mouse. For determining the relative amount of UDS and unrepaired nDNA SSB, nuclear grain numbers were microscopically analyzed for each of 100 consecutive cell profiles per cell type per animal (cells with zero grains were also included) (as done in [6], [13], [17], [18], [20], [21]) (Fig. 2A). Furthermore, the nuclear profile areas of these cells were recorded using a JVC KY-F75U digital camera (JVC, Tokyo, Japan) mounted on a DIAPLAN microscope (Leitz, Wetzlar, Germany) and DISKUS software (Hilgers, Königswinter, Germany). To measure the relative amount of mtDNA synthesis, grain densities per unit area of the perikaryal cytoplasm were analyzed for the same 100 cells per animal that were investigated for UDS. Cells in S phase (glial and endothelial cells in the brain sections as well as cells in kidney and liver) could be easily identified by their extremely high grain number above the nucleus (Fig. 2B). These cells were excluded from the evaluation. All microscopic analyses were carried out with a 100× oil immersion objective and a counting grid in the eyepiece with 400 small squares (6.0×6.0 µm^2^). Prior to statistical data processing, cumulated relative frequency (CRF) distributions of nuclear grain numbers (including zero grains) were constructed for each type of cell and animal (outlined in detail in [6]). This allowed for the identification of artifacts in the autoradiographs such as fading effects (visible on a statistically significant shift of a distinct CRF distribution curve to the left in comparison to the curves of equally treated mice) or an increase in background labeling (significant shift of a CRF distribution curve to the right). The corresponding autoradiographs were excluded from the analysis. Excluded autoradiographs could not be replaced by others because the method only allows for evaluation of autoradiographs from the same exposure box.

Mean silver grain numbers, obtained in the studies measuring the relative amount of UDS and mtDNA synthesis, were corrected for background (Fig. 2C) using Stillström's formulas [29], [30]. Corrections were done separately for each investigated type of cell using the grain numbers obtained on autoradiographs of the mice not injected with ^3^H-TdR. The latter autoradiographs were exposed in the same box as those from the animals injected with ^3^H-TdR.

Background of ISNT autoradiographs was also corrected using Stillström's formulas. Corrections were done using the grain numbers obtained on autoradiographs that were prepared in an identical manner to that described above, except without the application of DNA polymerase-I. These autoradiographs also clearly showed (in agreement with our previous study [6]) that the results of the ISNT analyses were not skewed by grains originating from the injected ^3^H-TdR done for the demonstration of UDS. This was due to the short exposure time of up to 12 days for ISNT autoradiographs, in contrast to the 250 day exposure for UDS autoradiographs.

Mean nuclear grain numbers were normalized in order to relate them to the actual nDNA content of the section volume [31], [32]. This was not carried out for the cytoplasmic labeling, as the number of mtDNA molecules per cell is not constant, but rather varies depending on the actual energy demand of the cell (c.f., e.g., [21]).

### Statistical Analysis

Comparisons of CRF distribution curves of grain numbers (outlined in detail above) were performed with the Kruskal-Wallis test. The CRF distribution curves of grain numbers that were statistically significantly different (i.e., p<0.05) from the corresponding CRF distribution curve of median grain numbers obtained for a group of equally pre-treated and evaluated mice were excluded from the analysis. This was done in the ISNT studies for two out of 30 mice.

For all groups of mice and all analyzed types of cells, mean and standard error of the mean (SEM) of the investigated variables (body mass, nuclear grain number, cytoplasmic grain density) were calculated. Comparisons between groups were performed with analysis of variance (ANOVA) for mean body mass and multivariate analysis of variance (MANOVA) for the other variables (general linear model; fixed factors: type of cell, flux density of the MF exposure; dependent variables: nuclear grain number representing UDS, nuclear grain number representing unrepaired nDNA SSB [ISNT analyses], cytoplasmic grain density representing mtDNA analysis). These variables were tested together because they depend on each other: less UDS may result in more unrepaired nDNA SSB, while less mtDNA synthesis can result in less ATP provision to energy-dependent nDNA synthesis (the basis of UDS; see, e.g., [33]). All calculations were carried out using SPSS (Version 21; IBM, Armonk, NY, USA). P-values smaller than 0.05 were considered to be statistically significant.

### Photography

The photomicrographs shown in Fig. 2 were produced by digital photography using a Zeiss AxioCam HRc digital camera (4,164×3,120 pixels; Carl Zeiss MicroImaging, Jena, Germany) attached to a Nikon Eclipse 50i microscope (Nikon Corporation, Tokio, Japan) and AxioVision software (version 4.8; Zeiss), using a 100× oil objective (numerical aperture  = 1.25). The final figures were constructed using Corel Photo-Paint ×6 and Corel Draw ×6 (both versions 16.1.0.843; Corel, Ottawa, Canada). Only minor adjustments of contrast and brightness were made using Corel Photo-Paint, without altering the appearance of the original materials.

## Results

No mice died during the 8-week MF exposure. The mean body mass of the animals did not differ between the three groups of mice at the end of the MF exposure (0 mT: 46.82±1.25 g (mean ± SEM); 0.1 mT: 47.53±1.25 g; 1.0 mT: 48.46±0.56 g; p_ANOVA_  = 0.476).

In the UDS analyses, autoradiographs from ^3^H-TdR-injected mice showed a five-to tenfold stronger signal than autoradiographs from control mice that did not receive ^3^H-TdR (Fig. 2). Likewise, for ISNT analyses, sections that were treated with DNA polymerase-I showed an approximately tenfold stronger autoradiographic signal than control sections that were not treated with DNA polymerase-I.

MANOVA showed statistically significant differences between the types of cells (ToC) (overall: p<0.001; UDS, unrepaired nDNA SSB, and mtDNA synthesis: p<0.001 each). With regard to the flux density (FD) of the MF exposure, MANOVA only showed statistically significant differences in mtDNA synthesis data between the investigated flux densities (overall: p = 0.016; UDS: p = 0.068; unrepaired nDNA SSB: p = 0.057; mtDNA synthesis: p = 0.012). For the combination of ToC and FD, MANOVA indicated statistically significant results for UDS and mtDNA synthesis (overall: p = 0.008; UDS: p = 0.001; SSB: p = 0.317; mtDNA synthesis: p = 0.001). Accordingly, the investigated MF exposure had no statistically significant impact on the relative amount of unrepaired nDNA SSB in the investigated types of cells.

**Figure 3:**
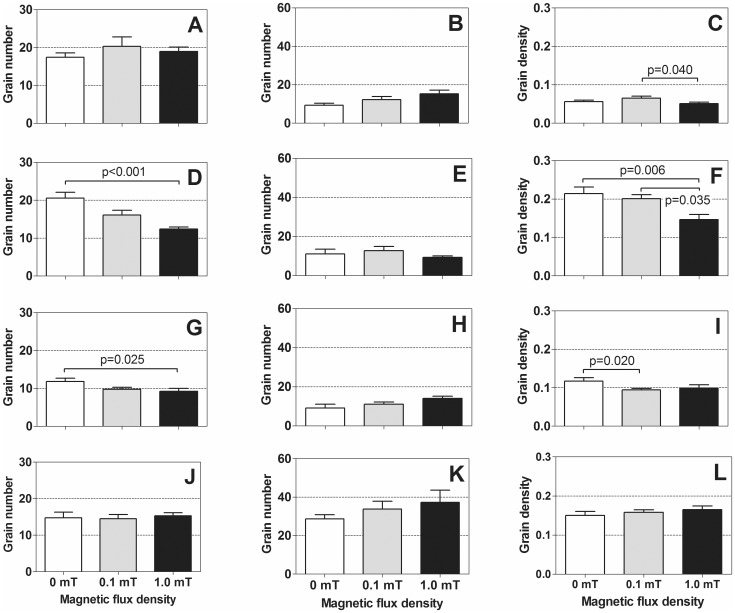
Results of the autoradiographic analyses. The graphs show mean and standard error of the mean (SEM) of grain numbers representing UDS (**A**, **D**, **G**, **J**), unrepaired nDNA SSB/ISNT (**B**, **E**, **H**, **K**) and mtDNA synthesis (**C**, **F**, **I**, **L**) of neurons in the caudate nucleus in the brain (**A–C**), epithelial cells in the choroid plexus of the fourth ventricle in the brain (**D–F**), epithelial cells of the cortical collecting duct in the kidney (**G–I**), and pericentral hepatocytes in the liver (**J–L**) after sham-exposure (open bars), MF exposure with 0.1 mT for eight weeks (gray bars), or MF exposure with 1.0 mT for eight weeks (closed bars). For a detailed description of the generation of these grain numbers and grain densities see Section “Evaluation of Autoradiographs” in the main text. Statistically significant differences between groups are indicated.

This observation was also reflected by the results of the cell-type specific analysis of UDS, unrepaired nDNA SSB, and mtDNA synthesis that showed only a few statistically significant differences between the groups of mice: (i) mean UDS was reduced after MF exposure with 1.0 mT compared to sham-exposure for both the epithelial cells in the choroid plexus of the fourth ventricle in the brain and the epithelial cells of the cortical collecting duct in the kidney (Fig. 3 D, G); (ii) neurons in the caudate nucleus in the brain showed reduced mtDNA synthesis after 1.0 mT MF exposure compared to 0.1 mT (Fig. 3C); (iii) epithelial cells of the choroid plexus of the fourth ventricle in the brain displayed reduced mtDNA synthesis after 1.0 mT MF exposure compared to both 0.1 mT MF exposure and sham-exposure (Fig. 3F); and (iv) epithelial cells of the cortical collecting duct in the kidney showed reduced mtDNA synthesis after 0.1 mT MF exposure compared to sham-exposure (Fig. 3I). The MF exposure had no statistically significant impact on the relative amount of unrepaired nDNA SSB in the investigated types of cells (Fig. 3B, E, H, K) and no statistically significant impact on the pericentral hepatocytes in the liver (Fig. 3J–L).

## Discussion

### Validity of the Results

Previous autoradiographic studies after injection of ^3^H-TdR into untreated adult Han: NMRI mice with an autoradiograph exposure time of 250 days resulted in similar mean grain numbers (representing UDS) and mean cytoplasmic grain densities (representing mtDNA synthesis) as found in the present study after sham-exposure [6], [13], [19]–[21]. Likewise, mean ISNT data obtained in the present study after sham-exposure were similar to those obtained in a previous study on control mice [13]. This confirms the validity of the results of the present study.

### Impact of MF Exposure With 0.1 mT on UDS, unrepaired nDNA SSB, and mtDNA synthesis

Except for slightly reduced mtDNA synthesis in the epithelial cells of the cortical collecting duct in the kidney, MF exposure of 0.1 mT over eight weeks had no impact on the UDS, relative amount of unrepaired nDNA SSB, or mtDNA synthesis of the investigated types of cells in the mouse brain, kidney, and liver. This finding is of great relevance to society as a magnetic flux density of 0.1 mT represents the current exposure limit for the general population in most European countries [22].

No definitive explanation could be found for the slightly reduced mtDNA synthesis of the epithelial cells of the cortical collecting duct in the kidney. Theoretically, the latter may be related to an effect of MF exposure on the enzyme thymidine kinase (TdR-K). This enzyme is necessary for phosphorylation prior to incorporating the injected ^3^H-TdR into DNA. The activity of TdR-K was found to be temporarily decreased following low-dose gamma-ray exposure [34], but increased after exposure to a stationary magnetic field of 1.4 T [35], [36].

### Impact of MF Exposure With 1.0 mT on UDS, unrepaired nDNA SSB, and mtDNA synthesis

Compared to sham-exposure, MF exposure with 1.0 mT resulted in statistically significantly reduced mean UDS of the epithelial cells in the choroid plexus of the fourth ventricle in the brain as well as in epithelial cells of the cortical collecting duct in the kidney. This could possibly be connected with reduced mtDNA synthesis in these types of cells (statistically significant for epithelial cells in the choroid plexus of the fourth ventricle in the brain following 1.0 mT MF exposure as compared to sham-exposure). Reduced mtDNA synthesis may represent reduced provision of ATP in the affected cells as nuclear DNA synthesis (the basis of UDS) is ATP-dependent (see, e.g., [33]). Thus, reduced mtDNA synthesis may also lead to reduced production of free radicals formed inside these cells, which is generally in line with ideas proposed by Lai & Singh [2]–[5]. This may also be reflected by the fact that none of the investigated cell types showed a statistically significant increase in relative amount of unrepaired nDNA SSB following continuous, eight week 50 Hz MF with a flux density of 1.0 mT as compared to sham exposure.

### Impact of MF Exposure With Magnetic Flux Density Higher than 1.0 mT on UDS, unrepaired nDNA SSB, and mtDNA synthesis

In our previous study [6], in which we exposed adult mice to continuous eight-week 50 Hz MF with a strong flux density of 1.5 mT, we found increased UDS and a higher amount of unrepaired nDNA SSB in the epithelial cells of the choroid plexus of the fourth ventricle in the brain, but not in pyramidal cells and glial cells in layer V of the cerebral cortex, endothelial cells in all cortical layers in the brain, granule and pyramidal cells in the hippocampus in the brain, and Purkinje and granule cells in the cerebellum [6] (note that the results of our previous study [6] (i.e., mean grain numbers and grain densities) cannot directly be compared to the results of the present study as the autoradiographs of these studies were exposed in different boxes). No changes in the rate of mtDNA synthesis were found in the various tissues. The increased amount of unrepaired nDNA SSB found in a specialized cell type in the brain (i.e., the epithelial cells of the choroid plexus) may be an effect of its iron transport, as generally hypothesized by Lai & Singh [2]–[5] as cause of DNA damage after MF exposure. Conversely, increased UDS and a higher amount of unrepaired nDNA SSB were not seen after continuous eight-week 50 Hz MF with a strong flux density of 1.5 mT in epithelial cells of the cortical collecting duct in the kidney in our previous study [6]. Because both plexus epithelial cells in the brain [7] and epithelial cells of the cortical collecting duct in the kidney [23] exhibit iron transport, it appears that cell involvement in iron transport is, on its own, not enough to predict sensitivity to damage by a strong MF exposure. This should be addressed in future studies. The societal relevance of this question may be limited, however, as this observation was made at a magnetic flux density that was fifteen times higher than the current exposure limit of 0.1 mT for the general population in most European countries [22].

### Conclusion

The detailed analysis of four different cell types in three different organs in the present study showed no evidence that a continuous MF exposure over two months with a flux density of 0.1 mT or 1.0 mT leads to persisting unrepaired nDNA SSB. Whether or not the cells were involved in iron transport (epithelial cells in the choroid plexus of the fourth ventricle in the brain and epithelial cells of the cortical collecting duct in the kidney) or iron storage (pericentral hepatocytes in the liver) did not prove to be a factor. Future studies on this topic may address other types of cells and different time points (days, weeks, or even months) after the end of the MF exposure.
